# T-ChroNet: Time-aware chromatin network reconstruction to detect dynamic regulatory programs in longitudinal epigenetic dataset

**DOI:** 10.1093/nargab/lqag034

**Published:** 2026-04-08

**Authors:** Stefano Di Giovenale, Ottavio Lischio, Clelia Cortile, Giacomo Corleone, Maurizio Fanciulli, Francesco Bonchi, Iros Barozzi

**Affiliations:** Gene Expression and Cancer Models Unit, Department of Research and Advanced Technologies Translational Research Area, IRCCS Regina Elena National Cancer Institute, Rome 00144, Italy; Department of Computer, Control and Management Engineering, Sapienza University of Rome, Rome 00185,Italy; Gene Expression and Cancer Models Unit, Department of Research and Advanced Technologies Translational Research Area, IRCCS Regina Elena National Cancer Institute, Rome 00144, Italy; Gene Expression and Cancer Models Unit, Department of Research and Advanced Technologies Translational Research Area, IRCCS Regina Elena National Cancer Institute, Rome 00144, Italy; Charles Darwin Department of Biology and Biotechnologies, Sapienza University of Rome, Rome 00185,Italy; Gene Expression and Cancer Models Unit, Department of Research and Advanced Technologies Translational Research Area, IRCCS Regina Elena National Cancer Institute, Rome 00144, Italy; Gene Expression and Cancer Models Unit, Department of Research and Advanced Technologies Translational Research Area, IRCCS Regina Elena National Cancer Institute, Rome 00144, Italy; Intesa Sanpaolo AI Research, Turin 10138, Italy; Center for Cancer Research, Medical University of Vienna, Borschkegasse 8a, Vienna 1090, Austria

## Abstract

Networks are widely applied to investigate relationships among individual components of complex biological systems. Recent application of biological networks, such as gene co-expression networks and gene regulatory networks, has been instrumental to define principles of transcriptional modulation in development and disease. However, computational methods that can embed the activity of *cis*-regulatory elements (CRE) into a network are still limited. Capturing temporal CRE activity within a network could help reveal regulatory programs involved in cell fate commitment and disease development. To address this, we present T-ChroNet (Time-aware Chromatin Network), a network-based method that models CRE as nodes and their temporal co-accessibility as edges. Through the detection of CRE sharing similar accessibility patterns over time, T-ChroNet allows the inference of putative upstream regulators and downstream biological pathways. We applied T-ChroNet to temporally-resolved CRE datasets, from both human and mouse, including chromatin accessibility (ATAC-seq) and histone post-translational modifications (H3K27ac ChIP-seq). T-ChroNet successfully recovered known regulators and enriched pathways for both modalities and species, while also uncovering novel putative factors and mechanisms regulating cell identity, organ development and disease progression.

## Introduction

*Cis*-regulatory elements (CRE) are non-coding elements that regulate spatiotemporal gene expression [1, 2]. Modulation of the activity of CRE is essential to execute cell-type-specific developmental programs, ensuring correct multi-cellular organism formation, while also responding to external stimuli. During early embryogenesis, CRE define the expression patterns promoting lineage commitment [3, 4]. At the same time, B-cells stimulated with IgG or Interleukin-4, exhibit rapid and dramatic changes in their chromatin landscape, with a marked and specific increase in CRE activity [5]. Epigenetic alterations or changes in the expression or activity of transcription factors (TF), also allow tumours to ectopically hijack CRE, enhancing the expression of pathways promoting survival and plasticity, in turn sustaining proliferation and cancer progression [6–8].

A large fraction of CRE is represented by enhancers and gene promoters. A basic model of enhancer activity includes the binding of TF and co-factors at locus-specific TF-binding sites, followed by an increase in the accessibility of the locus, production of non-coding RNA, and more frequent physical interactions between the CRE and the promoter of the target genes, thereby enhancing their transcriptional output [9, 10]. At active CRE, this process is epigenetically modulated via nucleosome spacing and post-translational modifications of the histone tails (e.g. H3K27ac, H3K4me1), which can be mapped by Chromatin Immunoprecipitation sequencing (ChIP-seq).

Gene co-expression networks and gene regulatory networks (GRN) have been widely and successfully employed to represent complex interactions among biological entities. Gene co-expression networks connect genes according to their similarity in RNA expression across different conditions or timepoints, in turn enabling the identification of modules of genes involved in similar pathways or cellular functions [11]. GRN expanded this concept to capture the direct, causal relationships between regulators (e.g. TF) and their target genes [12, 13]. Despite these advancements, currently available methods mainly focus on modelling the relations between genes or TF-genes without taking into account the pivotal role of CRE in regulating gene expression. To address this, the concept of enhancer Gene Regulatory Networks (eGRN) has been applied to both bulk [14, 15] and single-cell data [12, 13, 16, 17], extending classical GRN to incorporate CRE as intermediate nodes linking upstream regulators to their target genes. However, existing eGRN inference methods present notable limitations: they rely on the joint availability of multimodal data (RNA-seq and ATAC-seq), and their dependence on computational predictions of TF binding sites exposes them to a substantial rate of false positives [18–20]. Moreover, these approaches have yet to fully account for the temporal dimension of CRE activity. Indeed, while modelling temporal gene expression patterns has elucidated key regulatory events in development and disease [21, 22], analogous approaches to systematically characterize temporal modulation of CRE, especially enhancers, remain largely unexplored. However, accounting for the temporal modulation of CRE can yield significantly more insights into which upstream signals these CRE integrate, which in turn could be crucial to pinpoint developmental choices and malignant transformations. Therefore, computational methods to dissect the modulation of CRE in relation to temporal activity patterns remain insufficient.

To overcome this limitation, we propose T-ChroNet (Time-aware Chromatin Network), a network-based approach designed to define communities of CRE with consistent temporal activity patterns in longitudinal datasets. Each dataset is embedded into a network in which nodes are CRE and edges represent the accessibility correlation among the samples in the dataset. We applied T-ChroNet to the data from three distinct studies to demonstrate its wide applicability. Notably, for all the datasets considered, T-ChroNet consistently identified communities of CRE involved in similar biological processes and bound by subsets of TF consistent with their pattern in time. While results were in accordance with previous literature, T-ChroNet also pinpointed new downstream processes and upstream TF for several of the identified communities.

## Materials and methods

### T-ChroNet algorithm

T-ChroNet is a computational method to define communities of chromatin regions sharing similar patterns of accessibility (or chromatin state, as generally defined by histone modifications) along a temporal dataset. To address this, T-ChroNet models accessibility regions as nodes, with edges representing Spearman’s rank correlation coefficient between the two nodes’ accessibility patterns across the dataset. T-ChroNet takes as input a matrix (*n* x *m*) of log2-normalized counts, for *n* genomic regions, across *m* samples. Spearman’s rank correlation coefficients are calculated by breaking down all the possible pairs of nodes into chucks, and then parallelized. This largely limits RAM usage for large *n*. The memory usage can be controlled via two distinct parameters:

- Chunk size: the number of nodes processed in each parallel job. Smaller values lead to a higher number of parallel jobs, each considering a smaller number of nodes (lower memory usage); larger values lead to fewer iterations with a larger number of nodes (higher memory usage).
- Step size: this parameter controls the maximum number of pairwise correlations computed simultaneously during network construction. Correlations are evaluated in fixed-size batches (1 × 10⁵) rather than over the full chunk. This batching strategy limits RAM usage by freeing memory after each batch is processed. Larger step sizes reduce computational overhead at the cost of higher memory usage, whereas smaller step sizes reduce memory requirements but increase computational time.

Spearman’s rank correlation coefficients are computed using a rank-based transformation of the signal intensity input matrix, followed by correlation calculation. Specifically, the input feature matrix is first converted to rank across samples through scipy.stats.rankdata() function (‘average’ method). For each row (feature), intensity signals are independently ranked across samples. Then, the rank-transformed values are standardized on a per-feature basis by subtracting the mean rank and dividing by the standard deviation across samples. The normalization step is implemented using NumPy array operations to ensure that each feature has a zero mean and unit variance after ranking. Pairwise correlations are then computed as the normalized dot product between standardized rank vectors using NumPy operation *einsum*. If specified, statistical significance is assessed using SciPy *t*-distribution functions, and correlations not meeting the specified *p*-value threshold are discarded prior to network construction.

The core T-ChroNet algorithm is implemented as a Python package, named TChroNetPy. It generates the correlation graph through the multicore process described above. During this step, the user can specify either a lower-bound threshold on the Spearman’s rank correlation coefficient or on the *p*-value of the correlation coefficient. In the latter case, the minimum correlation threshold was determined by calculating the critical *t*-value corresponding to the target *p*-value using Student’s *t*-distribution (SciPy). This *t*-statistic was then transformed into a correlation coefficient based on the number of samples (degrees of freedom). From this, a minimum correlation threshold was established; coefficients that did not meet this threshold were excluded from the study. The lower Spearman’s rank correlation coefficient that still meets the user-specified threshold is then considered as a lower-bound threshold for a correlation to be considered. To reduce memory usage, correlations are computed and edges stored considering stepwise intervals of 0.1 (e.g. 0.4–0.5, 0.5–0.6, and so on). Correlation values are stored in a DeepGraph object, which acts as an edge manager, keeping only significant edges in memory and writing them to disk in Parquet files. This approach enables the construction of large feature-feature networks while limiting RAM usage and producing final edge lists organized by correlation strength.

In addition to TChroNetPy, we developed TChroNetR, an R library designed to facilitate downstream analysis of the resulting networks. Parquet edge files, divided into correlation-based chunks, are read and stored in a *TChroNetSeries* object, along with multiple network metrics (number of edges, number of nodes, density, transitivity, largest connected component, or LCC, and relative LCC, or rLCC) for each network defined by different thresholds on Spearman’s rank correlation (see above). By utilizing the data stored within a *TChronetSeries* object, users can identify the optimal threshold for further analysis. The process of network selection can be manually curated by the user or automatically determined by TChroNetR. The automatic routine selects the threshold that yields the highest value of rLCC, favouring well-connected networks. Importantly, all nodes from the original feature matrix are preserved in the network, even if they are disconnected, ensuring that isolated genomic regions are retained for subsequent steps of the analysis. In case of equal rLCC values among different thresholds, the algorithm favours the more stringent threshold (i.e. higher correlation values), effectively removing low-value edges that do not alter the network topology. During the definition of a *TChronetSeries* object, the user can set a range of resolution parameters for community detection (Leiden algorithm [23]). In this way, the selection of the optimal correlation threshold can be further manually guided by investigating the stability of the communities detected at increasing resolutions (see below) in the context of the increasing thresholds imposed on the graph. The nodes in the disconnected components can be either discarded or considered as part of a separate, additional community. This can be tuned as a parameter in TChroNetR, so that the user has the opportunity to include the additional community in the downstream annotation steps.

For community detection analysis and downstream analyses to ease biological interpretation of the communities, the *TChroNetSeries* object is transformed into a *TChroNetNetwork* object. Since the community detection process is highly dependent on the resolution parameter, we have included an automatic resolution parameter selector based on modularity. Modularity measures the extent to which nodes within a community are more densely connected to each other than to nodes in other communities. By maximizing modularity, the algorithm favors partitions that capture strongly interconnected groups of features, ensuring that the detected communities reflect meaningful network structure rather than arbitrary subdivisions. This approach improves the biological interpretability of the resulting communities and reduces user bias in selecting the resolution parameter. TChroNetR also provides functions to perform biological annotation through automatic detection of target genes using rGREAT [24] , a lift-over function from rtracklayer R library (v. 1.66.0) [25] that converts coordinates between different genome versions, and functions to annotate the genomic regions according to the SCREEN compendium [26] (or a user-specified BED file), and for inferring upstream transcription regulators using the MonaLisa R library [27], for each community.

### T-ChroNet analysis of H3K27ac profiles from THP-1 cells

Raw counts were downloaded from the original publication [28] and normalized through the R package edgeR [29] (v 4.4.2). Peaks were filtered through the edgeR function filterByExpr(), with default parameters. Finally, the counts were normalized using the cpm() function with log = TRUE. A batch correction step was then applied using the R package limma (v3.62.2; removeBatchEffects() function).

Peaks annotated as “STATIC” by the authors [28] were removed, together with peaks on chromosomes X and Y. Normalized counts from biological replicates corresponding to the same time point were averaged, and the resulting mean normalized peak counts were used for downstream analyses.

To calculate the Spearman’s correlation, we employed TChroNetPy using as input the normalized matrix and with parameters: –min_t_type pval –min_t_val 0.1. The resulting files were analysed through the TChronetR library. The Parquet files were loaded into a *TChroNetSeries* object, and a threshold of 0.8 on the edge weights was selected according to the rLCC. To identify the best resolution parameter for Leiden community detection [23], we evaluated the resulting communities, setting resolution from 0.5 to 1.5, with a 0.1 step. Modularity was computed for each resolution, and the resolution value of 1.0 was selected as it maximized it.

The regions in the detected communities were compared to those in the clusters detected in the original paper [28], and the overlapping percentage was evaluated.

In order to compare trends across regions and communities, normalized counts per region across time points were transformed using a *z*-score.

For each community, a set of nearest genes was defined as described in the section *Characterization of the identified communities*. Raw gene expression counts of genes showing differential expression in at least one time point were derived from the original publication [28]. Counts were transformed to CPM, and then log-normalized, using the R package edgeR [29] (v 4.4.2). In order to compare trends across genes, *z*-score standardization was applied.

### T-ChroNet analysis of ATAC-seq profiles from the developing mouse liver

For each sample, BAM files with the aligned reads and BED files with called peaks were downloaded from the ENCODE DCC [30]. To define a list of sites showing a significant signal in at least one sample, BED files were concatenated and merged with bedtools merge (v2.30.0) with default parameters. Regions mapping to X and Y chromosomes were excluded. Raw counts were then estimated over each region and separately for each sample using bedtools multicov. Filtering of extremely low and high signals, as well as normalization, was performed as described in the previous paragraph. Regions with low variance along the analysed cohort were then excluded by removing those with variance lower than 0.1. Finally, the mean between replicates of the same time point was calculated. The resulting matrix was passed to TChroNetPy with parameters: –min_t_type pval –min_t_val 0.1. The resulting files were loaded into a *TChroNetSeries* object, and a threshold of 0.8 on the edge weight was selected according to the rLCC. To identify the best resolution parameter for Leiden community detection [23], we evaluated the resulting communities, setting resolution from 0.5 to 1.5, with a 0.1 step. Modularity was computed for each resolution, and the resolution value of 1.1 was selected as it maximized it.

### T-ChroNet analysis of ATAC-seq profiles from BCP-ALL

FASTQ files were downloaded from the GEO (GSE214916) and analysed through the Nextflow [31] nf-core ATAC-seq pipeline with parameter –macs_gsize 2864785220. The raw count matrix generated by the pipeline on a consensus peak set was normalized with the R package edgeR [29] (v4.4.2). Regions on chromosome X and Y were filtered out, along with peaks showing a variance lower than 1.0. Filtering of extremely low and high signals, as well as normalization, was performed as described in the previous paragraph. To identify the optimal threshold on correlation, we run TChroNetPy with parameters: –min_t_type pval –min_t_val 0.1. The resulting files were loaded into a *TChroNetSeries* object and a threshold of 0.5 on the edge weight was selected according to the rLCC. To identify the best resolution parameter for Leiden community detection [23], we evaluated the resulting communities, setting resolution from 0.5 to 1.5, with a 0.1 step. Modularity was computed for each resolution, and the resolution value of 1.0 was selected as it maximized it.

### Characterization of the identified communities

#### Ontologies

To detect a set of target genes for each community, we used the R package rGREAT [24] (v 2.8.0) through the submitGreatJob() function with version = 4.0.4 and rule = basalPlusExt. The resulting genes were employed to interrogate MSigDB through the R package GSEABase (v 1.68.0). Enriched terms were obtained through the enricher() function from the R package clusterProfiler (v. 4.14.0) using the complete list of nodes as background. Resulting terms were filtered according to *q*-value < 0.05 (for THP-1 and Liver development datasets) and *p*-value < 0.01 (for BCP-ALL dataset). For each dataset, we used a different, more suitable reference database: Hallmark [32] (H) for the THP-1 dataset, Mouse Cell Type Signature [33] (M8) for the Mouse Liver Development dataset, and Curated Cancer Cell Atlas gene sets [34] (C4:3CA) for the BCP-ALL patients dataset.

The top ontologies were selected to create a set of enriched ontologies, and enrichment score and -log10(*q*-value) were used for visualization purposes.

#### Transcription factor detection

The regions in each community were converted from hg19 to hg38 (for human datasets) using the lift_network_coordinates() function of the TChroNetR library. The conversion between hg19 and hg38 was performed using the UCSC [35] hg19-to-hg38 chain file as input for the function. Converted coordinates were used to query the CistromeDB database [36] web server and obtain the putative TF enriched in the regions of each community. CistromeDB provides enrichment scores in the form of GIGGLE scores [37], derived from overlaps with available ChIP-seq experiments. A GIGGLE score takes into account both the significance and the magnitude (effect size) of the overlap (it equals -log10(*p*-value) * log2(odds-ratio)). Since multiple ChIP-seq experiments may be available for the same TF, the provided GIGGLE scores were aggregated by computing the mean score for each TF across all the corresponding experiments. Transcription factors were then ranked according to their mean GIGGLE scores, and the top ten TF for each community were retained for visualization.

We performed genomic annotation for the regions in each community via the annotate_regions_from_bed() function in the TChroNetR library. To annotate the peaks, we downloaded the annotation files from SCREEN [26] for hg38 and mm10. The resulting annotations were used to evaluate the fraction of regions per community mapping to different types of genomic regions.

### Running time evaluation

T-ChroNetPy running time was evaluated for every possible combination of number of cores (1, 2, 5, 15, 20, 25, 30, 35, 40) and node count (1 000, 5 000, 10 000, 20 000, 40 000, 60 000, 80 000, 100 000). During each iteration, a random subset of accessible regions was chosen from the BCP-ALL normalized counts table. To remove spurious correlation, we ran TChroNetPy with parameters –min_t_type pval –min_t_val 0.1.

### Benchmark against TCSeq and WGCNA

To evaluate the performance and scalability of our computational framework, we conducted a comprehensive benchmark comparing TCseq (using both *k*-means and hierarchical clustering), WGCNA, TChroNetPy, and TChroNetR. To ensure a fair comparison, all methods were tested using identical peak sets randomly sampled from the developing mouse liver dataset.

#### Experimental design and scalability

We assessed how each method scales by testing them across datasets of increasing complexity, specifically: 1k, 5k, 10k, 20k, 40k, 60k, 80k, and 100k peaks. For every trial, we monitored peak RAM consumption and total execution time to quantify hardware efficiency. Notably, WCGNA and TCseq failed to analyse datasets larger than 60 000 regions, with WGCNA failing due to an out-of-memory error, and TCSeq due to a memory-related error occurring during silhouette calculation.

#### Individual configuration of the tools

We tailored the settings for each tool to ensure optimal performance:

- WGCNA: raw counts were loaded, then a soft-thresholding approach was applied based on WGCNA’s internal automated selection. Clustering was performed with the function cutreeDynamic() with minClusterSize set to either 100 or 1000.
- TCseq: BED and BAM files for count evaluation were provided. Using standard parameters, we applied an automatic silhouette maximization via the fviz_nbclust() function in the R package cluster (v. 2.1.8.1). This procedure was employed to identify the optimal number of clusters for both k-means and hierarchical clustering approaches.
- TChroNetPy: a p-value threshold (–min_t_type pval –min_t_val 0.1) to filter for significant interactions was applied.
- TChroNetR: a TChroNetSeries object was used to automatically identify the optimal correlation threshold. After converting the data into a TChroNetNetwork object, we performed community detection across resolutions ranging from 0.5 to 1.5 (with a 0.1 increment).

#### Biological interpretation

To validate the biological relevance of the findings, we compared the results from TCseq, WGCNA, and TChroNetR. For this comparison, we focused on the communities detected by TChroNetR at a resolution of 1.1. For both TCseq and WGCNA, we used silhouette maximization to define the optimal number of clusters. We then performed enrichment analysis for the identified communities, following the protocols described in the *Characterization of the identified communities* paragraph. Transcription factor analysis was conducted using the R package monaLisa (v1.12.0) (background = “genome” and genome = “BSgenome.Mmusculus.UCSC.mm10” (v. 1.4.3) paired with the JASPAR2024 database (tax_group = “vertebrates,” collection= “CORE,” matrixtype = “PWM,” all_versions = FALSE). To ensure high-confidence results, we only retained TF with a -log10(*p*-value) > 4 and a log2(enrichment) > 1. The top 20 TF were retained for downstream analysis and plotting.

## Results

### T-ChroNet workflow

**Figure 1.**
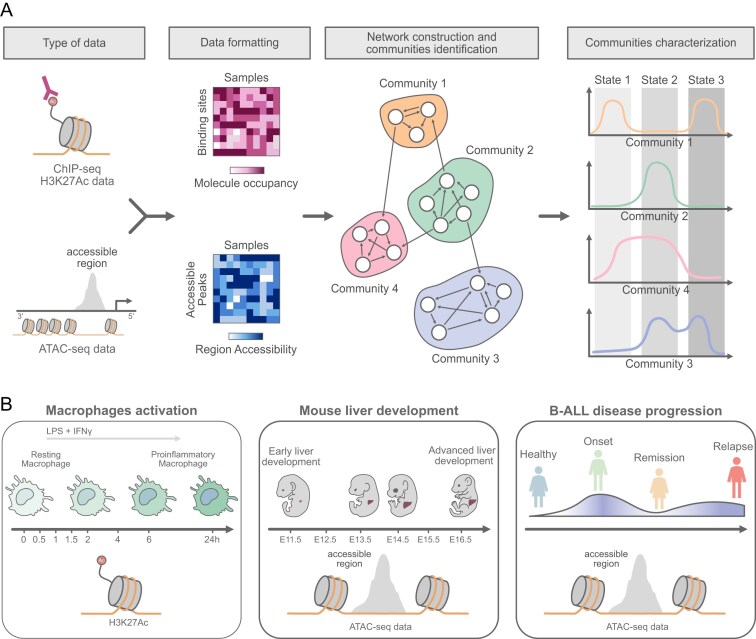
The T-ChroNet workflow. (**A**) Schematic summarizing the computational workflow. ATAC-seq or H3K27ac ChIP-seq data are processed into a matrix with the identified accessible or modified regions on the rows, samples on the columns, and values representing log2-normalized counts. The resulting matrix is then employed to build a chromatin network in which nodes are the regions, and edges connect pairs of regions with a Spearman’s correlation coefficient exceeding a defined threshold. Graph-based community detection algorithms are then employed to detect groups of co-regulated regions (community), which capture distinct temporal trends. (**B**) Cartoon showing the three datasets employed: (left) Macrophages activation through IFNγ and LPS stimulation, showing temporal chromatin changes, profiled by H3K27ac ChIP-seq; (middle) Mouse liver development from stages E11.5 to E16.5, profiled through ATAC-seq; and (right) B-ALL disease progression in which accessibility profiles were obtained from patients at four consecutive stages (healthy, primary, remission, relapse), by ATAC-seq.

T-ChroNet is a computational framework designed to identify communities of non-coding genomic regions that exhibit similar accessibility dynamics (or predicted activity dynamics, based on other -omics readout) over time (https://github.com/DGStefano/T-ChroNet). By leveraging an undirected weighted co-accessibility network approach, T-ChroNet captures regulatory relationships between genomic regions, offering a powerful tool to analyse chromatin accessibility changes in both development and disease-related biological processes (Fig. 1A).

T-ChroNet can model biological signals from cell lines, primary tissues, and human patient-derived samples profiled through either assay for transposase-accessible chromatin with high-throughput sequencing (ATAC-seq) or histone marks. Notably, T-ChroNet can embed other information, such as clinical annotations of the samples, into the network, allowing a more narrowed stratification of the accessible sites analysed. The multi-thread implementation of T-ChroNet can build networks with 40 000 nodes in ∼10 min when using at least two threads (Supplementary Fig. S1A). Importantly, T-ChroNet also includes functions to explore the sensitivity of the results to different values of the parameters imposed, and to automatically optimise them (see Methods).

To assess its performance, we applied T-ChroNet to three publicly available datasets, demonstrating its broad applicability. In all cases, communities mined by T-ChroNet show high consistency when upstream TF and gene ontologies (GO) associated with nearby target genes are computationally evaluated for each community of CRE identified from the network.

### T-ChroNet recapitulates the temporal trends of CRE activation in IFNγ-stimulated cells

We tested T-ChroNet on a temporal dataset of THP-1 cells stimulated with IFNγ and lipopolysaccharide (LPS) [28] (Fig. 1B, left). Treated cells have been collected at eight time points after stimulation (0, 30, 60, 90, 120, 240, 360, and 1440 min) and profiled to investigate the evolution of 3D chromatin structure, enhancer activity (via histone post-translational modifications), and gene expression during time progression. To validate T-ChroNet, we normalized the available H3K27ac ChIP-seq counts of time-modulated putative enhancers (as previously described by the authors), excluding those annotated as static (*n* = 43 446). Samples show consistency between replicates in PCA (Supplementary Fig. S2A) and a high correlation between samples and replicates of consecutive time points (Supplementary Fig. S2B). TChroNetR generated a co-accessibility network consisting of 43 446 nodes and ∼81M edges with a density of 0.086, after removing all Spearman’s correlations below 0.8. The threshold on Spearman’s correlation was automatically evaluated by TChroNetR, which identified the optimal cutoff according to the relatively largest connected component.

To evaluate the best resolution parameters according to the network topology, we evaluated the modularity and community composition at increased resolution. Modularity [38] was used to evaluate the strength of each network partition into communities, with values ranging from 0.5 to 1.5, corresponding to poor and good network separation, respectively. A Leiden resolution of 1.0 was automatically selected by TChroNetR according to the modularity score obtained at increased resolution. This parameter was selected since it achieves a robust community structure, while maintaining a fine level of granularity (Supplementary Fig. S2C).

We then assessed the robustness of these results, not only at varying resolutions on community detection but also by increasing or decreasing the minimum threshold applied to the Spearman’s correlation coefficient, in order for an edge to be retained (see Methods). Changing the parameters led to very similar communities (Supplementary Fig. S2D and E), with the exception of further fragmentation at correlations above 0.9.

**Figure 2.**
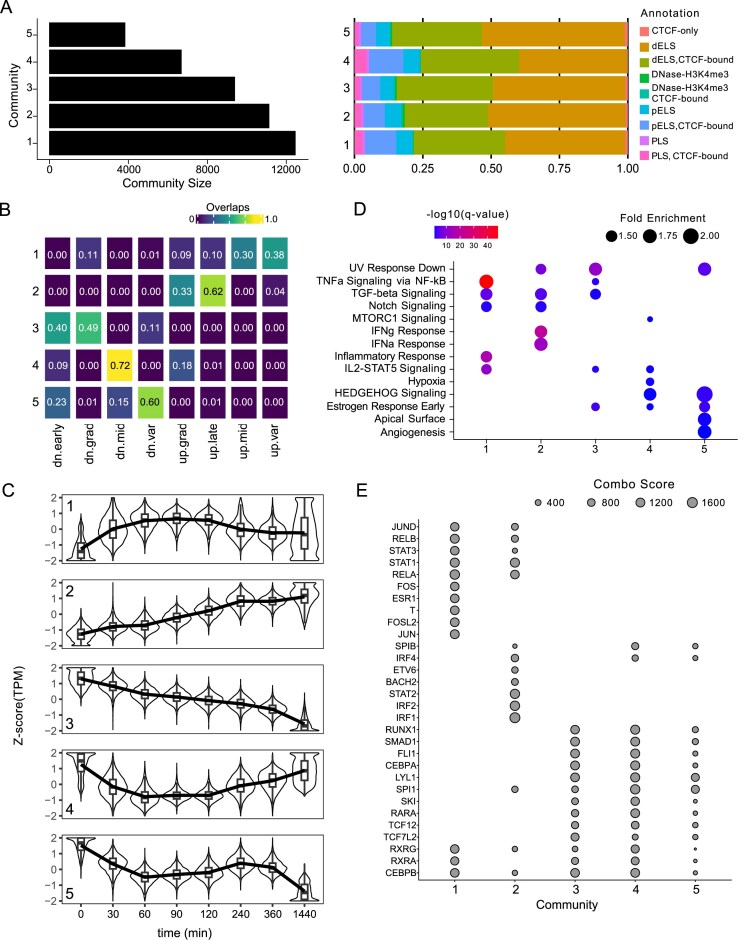
T-ChroNet analysis of THP-1 cells stimulated through INFγ and LPS. (**A**) (Left) Bar plot depicting the number of nodes forco each detected community. (Right) Stacked bar plot showing the percentage of regions associated with each genomic annotation, according to the SCREEN database, for each community. PLS: Promoter-Like Signature; pELS: Proximal Enhancer-Like Signature; dELS: Distal Enhancer-Like Signature. (**B**) Heatmap displaying the percentage of sites overlapping the temporal annotations provided by the authors (columns) and communities detected by T-ChroNet (rows). The number in each cell of the heatmap indicates the overlapping fraction. (**C**) Box plots overlaid on violin plots, showing the temporal dynamics signals (*z*-score [TPM]) across the indicated communities (number in the left corners of the plots). Each panel represents one community, with the *y*-axis representing the *z*-score (TPM) and the x-axis indicating the time points after stimulation (in minutes). Box plots show the median and the interquartile range. Medians of each consecutive box plot are connected by a line to highlight the temporal trend. (**D**) Bubble chart summarizing the enrichment for the Hallmarks gene sets, detected in the target genes of the CRE in each community. Bubble size represents the fold enrichment, while the colour indicates the -log10(*q*-value) (Hypergeometric test; Benjamini–Hochberg correction). (**E**) Bubble chart summarizing the top five transcription factors (*y*-axis) enriched in each community (*x*-axis). The size of the points indicates the Giggle Score.

At the optimally defined parameters (correlation of 0.8 and resolution of 1.0, see above), T-ChroNet identified five communities with more than 2000 nodes each (Fig. 2A, left). As expected, genomic annotation of the regions in each community highlighted a dominance of TSS-distal regions in spite of promoter-TSS (Fig. 2A, right). We then compared our communities with the clusters provided by the authors. While based solely on the temporal dynamics of the H3K27ac signal, T-ChroNet broadly recapitulates the annotations derived by the authors (which also relied on genome organization and gene expression), while avoiding mixing of regions with opposite trends (Fig. 2B). We then faceted the input regions by cluster and community (Supplementary Fig. S2F and G). While this confirmed the general consistency, T-ChroNet tends to redistribute small subsets of regions from the original clusters by incorporating them into distinct communities that show a more coherent temporal trend. For example, while the majority of the up-grad regions are captured by community 2, a small fraction of the regions is redistributed to either community 1 (which shows a general early upregulation) or community 4 (which shows transient downregulation; Supplementary Fig. S2F and G). On top of this, T-ChroNet shows a tendency to merge clusters, rather than splitting them. For example, community 1 captures regions that are up-regulated early, incorporating some showing a consistent pattern but that in the original paper were distributed across several, less distinctive groups (up-mid and up-var; Supplementary Fig. S2F and G).

We further assessed the temporal modulation of each community, resulting in five distinct patterns of H3K27ac signals over time (Fig. 2C and Supplementary Fig. S2I and J). The temporal activation or deactivation pattern is also followed by the genes putatively regulated by regions in each community. Indeed, by linking each enhancer to the nearest genes (considering those differentially expressed in at least one time point), we observed a consistent but temporally delayed change in expression of the putative target genes, as compared to the relative trend of H3K27ac of the community (Supplementary Fig. S2H). This observation reinforces the ability of T-ChroNet to retrieve groups of regions with meaningful co-modulation in time.

By investigating the Hallmarks [32] associated with the target genes in each community, we confirmed the robustness of T-ChroNet’s communities (Fig. 2D). Indeed, terms related to response to interferon were enriched in community 2, which is composed of enhancers with a gradual increase in activity over time, with NF-kB signalling enriched in community 1 (early upregulated) instead. Conversely, communities 3 and 5, representing early accessibility decrease and bimodal (early and late) accessibility decrease, respectively, showed enrichment for oestrogen response, which is known as an anti-inflammatory signal [39].

We then investigated the putative TF bound to the considered CRE, for each community, using annotations in CistromeDB [36] (Fig. 2E). Notably, highly scoring TF in community 2 included IRFs, NFKB1, and STATs, which are TF involved in the IFNγ response. Community 1 enriched for members of the AP1 family (JUN, FOS, JUND, FOSL2) that are known to be involved in the primary response. Conversely, communities 3 and 4 were enriched for transcription factors (TF) mainly involved in myeloid cell commitment, such as PU.1 (SPI1) and the more specific macrophage gene expression modulators CEBPA and CEBPB [40]. The enrichment of these TF could be due to a pro-inflammatory effect that induces a specific response in THP-1 cells through the modulation of enhancers bound by cellular fate commitment TF. Our results, in accordance with a previous study, also identified STAT1 as one of the most-enriched TF in community 1, a community of regions that exhibit a pronounced peak of activity around 60 min after the stimulation [41], consistent with a role of STAT1 in early transcriptional regulation response to IFNγ.

In conclusion, T-ChroNet identifies groups of CRE showing distinct temporal patterns of H3K27ac, enabling the interpretation of temporal modulation of enhancers profiled through ChIP-seq. In line with this, communities defined by T-ChroNet are biologically consistent according to the functional annotation of their nearby genes and the upstream TF enriched in their CRE.

### Dissection of the temporal evolution of different populations in the developing mouse liver using T-ChroNet

We applied T-ChroNet to an ATAC-seq time course from foetal mouse livers (profiled at stage E11.5, E12.5, E13.5, E14.5, E15.5, and E16.5) from ENCODE [42] (Fig. 1B, central). By first examining the whole accessibility landscape of two replicates (by PCA and correlation analyses), we found duplicates of the developmental stage to be highly similar. Additionally, samples from adjacent stages exhibit higher similarity than those from more distant from each other, supporting robustness of the data, and suggesting a smooth change in accessibility landscape over time (Supplementary Fig. S3A and B). To reduce the number of peaks in the network reconstruction step, we considered only those peaks showing high variability across samples.

**Figure 3.**
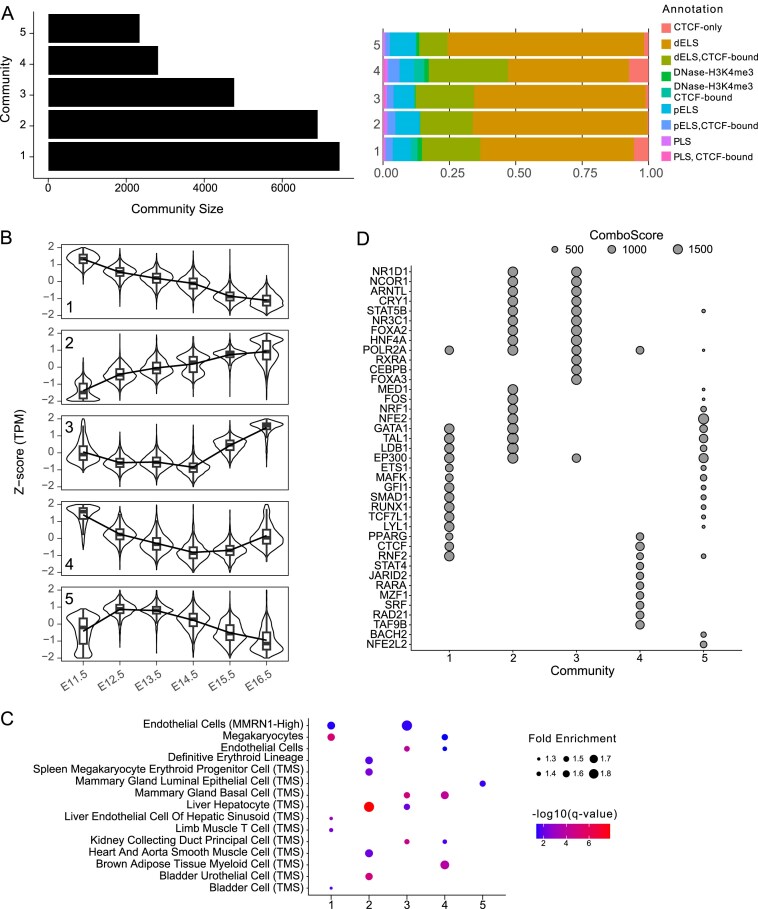
T-ChroNet analysis of Mouse liver development. (**A**) (Left) Bar plot depicting the number of nodes in each community. (Right) Stacked bar plot showing the percentage of regions associated with each genomic annotation, according to the SCREEN database, for each community. PLS: Promoter-Like Signature; pELS: Proximal Enhancer-Like Signature; dELS: Distal Enhancer-Like Signature. (**B**) Box plots overlaid on violin plots, showing the temporal dynamics signals (*z*-score [TPM]) across the detected communities. Each panel represents one community, with the *y*-axis representing the *z*-score (TPM) and the *x*-axis indicating the time points. Box plots show the median and the interquartile range. Medians of each consecutive box plot are connected by a line to highlight the temporal trend. (**C**) Bubble chart representing the enrichment for markers of specific cell types (mouse MSigDB datasets) in the target genes of the CRE in each community. Bubble size represents the fold enrichment, while the colour indicates the -log10(*q*-value) (Hypergeometric test; Benjamini–Hochberg correction). TMS: Tabula Muris Senis. (**D**) Bubble chart depicting the top 20 transcription factors (*y*-axis) enriched in each community (*x*-axis). The size of the points indicates the Giggle Score.

In a nutshell, after batch correcting and normalizing the raw counts, peaks with variance lower than 0.1 were removed. The network built by T-ChroNet on 22 486 accessible regions showed ∼34M edges (Spearman’s Correlation ≥ 0.8 according to the automatic T-ChroNet threshold selection), and a density of 0.14. To determine the best resolution parameter to conduct community detection, we systematically assessed both modularity and community stability across a range of resolution parameters (0.5–1.5). Based on both modularity optimization and the stability of communities across a large range of resolutions (Supplementary Fig. S3C), we selected a resolution parameter of 1.1, which resulted in five distinct communities (Fig. 3A). Similar to the analysis of THP-1 data, changing the parameters led to very similar communities (Supplementary Fig. S3D and E), with the exception of further fragmentation at correlations above 0.9. The identified communities showed distinct accessibility trends throughout the considered stages of mouse liver development (Fig. 3B and Supplementary Fig. S3F and G). These might reflect dynamic regulatory changes in specific cell types that can influence cellular differentiation, functional specialization, and tissue maturation. To test this, we evaluated the enrichment for cell type signatures annotated in MSigDB [33] in the target genes of each community, along with the TF enriched in their CRE. The annotation of the resulting communities is consistent with the liver being a transient site of haematopoiesis during mid-late gestation and gradually switching to metabolic functions at birth [43]. In line with this, community 5, which spans accessible sites with an early increase in accessibility during mid-gestation, showed enrichment for hematopoietic TF TAL1 [44], LDB1 [45] and GATA1 [46] (Fig. 3D). Community 1 instead showed a downwards trend in chromatin accessibility over time, in line with the enriched TF and ontologies (Fig. 3C and D). Community 1 was indeed dominated by repressive signatures, and TF of megakaryocyte cell commitment, such as RUNX1 [47] and GATA1 [48, 49]. Communities 2 and 3 appeared instead to predominantly capture sites accessible at later stages. In line with this pattern, this community is strongly enriched for signatures and TF related to hepatocytes and cholangiocytes specification and maturation, including HNF4A [50] and FOXA2 [51], respectively.

Taken together, these results highlight the ability of T-ChroNet to handle ATAC-seq data and to find communities of CRE showing similar behavior over time, in turn allowing the biological interpretation of each community also in the context of complex, heterogenous tissues whose cell-type identities and composition are rapidly changing through organism development.

### T-ChroNet enables co-accessibility analysis of CRE in heterogeneous cancer samples

**Figure 4.**
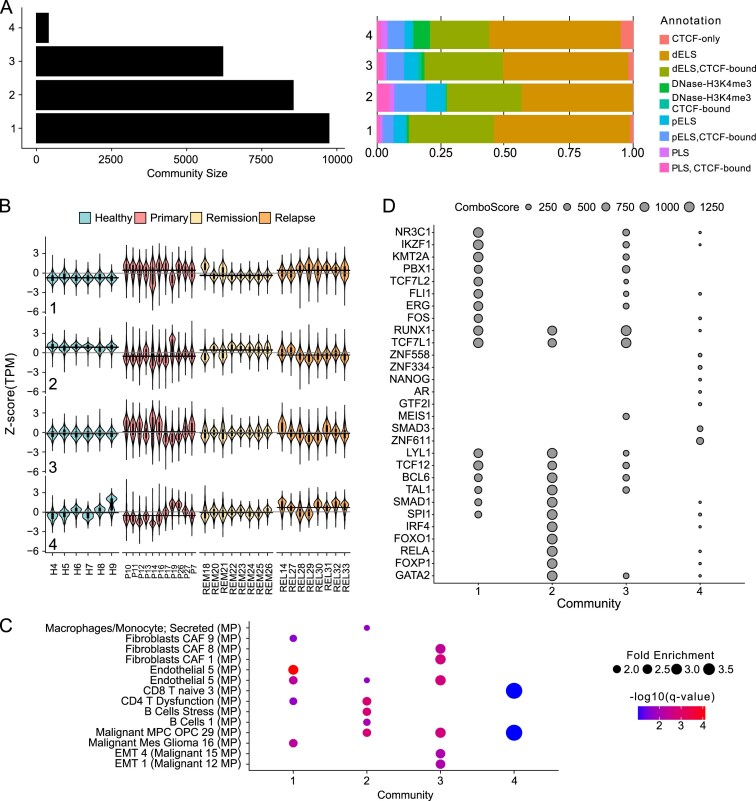
T-ChroNet analysis of B-ALL cancer progression. (**A**) (Left) Bar plot depicting the number of nodes for each community. (Right) Stacked bar plot showing the percentage of regions associated with each genomic annotation, according to the SCREEN database, for each community. PLS: Promoter-Like Signature; pELS: Proximal Enhancer-Like Signature; dELS: Distal Enhancer-Like Signature. (**B**) Violin plots depicting the distribution of accessibility signals are shown as *z*-score (TPM) for each detected community. Each panel divides patients according to the clinical annotation. Inside each violin, a boxplot represents the median and interquartile distribution. Black solid indicates the median accessibility across patients with the same clinical annotation. (**C**) Bubble plot representing the enrichment of Cancer Modules from human MSigDB datasets detected in genes linked to CRE in each community. Points’ size represents the Fold enrichment, and the colour represents the -log10(*q*-value) (Hypergeometric test; Benjamini–Hochberg correction). MP: Meta Program. (**D**) Bubble plot depicting the top 20 transcription factors (*y*-axis) enriched in each community (*x*-axis). The size of the points represents the Giggle Score.

Next, we applied T-ChroNet to identify communities from accessible regions mapped by ATAC-seq and potentially modulated during paediatric B-ALL cancer progression [52]. Despite the considerable inter-patient heterogeneity (Supplementary Fig. S4A and B), the substantial temporal differences between samples, and the lack of direct patient-matched longitudinal samples, T-ChroNet was able to detect communities of accessible chromatin regions that exhibit temporally homogeneous modulation throughout cancer progression. The analysed cohort consists of 32 samples enriched for CD19 + cells, each obtained from a different patient, spanning four distinct stages of cancer progression: Healthy, Pre-Treatment, Remission Post-Treatment, and Relapse (Healthy = 6, Pre-Treatment = 11, Remission Post-Treatment = 8, and Relapse = 7) (Fig. 1B, right). We first removed regions showing low variance across the whole cohort (Supplementary Fig. S4C). The resulting regions (*n* = 24 999) were used to build the co-accessibility network with ∼32M edges, and a density of 0.10. Modularity analysis and community stability at different resolution parameters pointed to 1.0 as the best resolution parameter (Supplementary Fig. S4D). Similar to the analysis of the other datasets, changing the parameters led to very similar communities (Supplementary Fig. S4E and F), with the exception of further fragmentation at correlations above 0.8. Taken together, T-ChroNet detected four communities of CRE (Fig. 4A).

Despite the high inter-patient heterogeneity, each community captured a distinct temporal pattern of chromatin accessibility within the cohort, including both patient-specific and disease-specific trends (Fig. 4B and Supplementary Fig. S4G). T-ChroNet detected two main communities, one showing an overall increase in accessibility in disease-like samples (Pre-Treatment and Relapse) and one with higher accessibility in healthy-like samples (Healthy and Remission), respectively, communities 1 and 2. To improve interpretation, the identified communities were systematically inspected through the lenses of the Cancers modules (CM) associated with the target genes identified in the vicinity of the CRE in each community (Fig. 4C). The community with higher accessibility in disease-like status (community 1) showed enrichment for CM-linked endothelial cells, supporting the involvement of these non-coding regions in malignant transformation [34] Conversely, communities in which accessibility is higher in healthy-like status enriched for CM of normal development of B-cells. Further analyses were conducted to identify and characterize the TF that are predominantly enriched within each community. This was achieved through a systematic interrogation of CistromeDB, allowing us to pinpoint key regulatory factors that may drive the observed chromatin accessibility patterns (Fig. 4D). Consistent with previous findings, Community 1 was highly enriched in TF known to play a pivotal role in B-ALL transformation, including RUNX1, ERG, and BCL6. In contrast, IRF4, GATA2, FOXO1, and SPI1 are among the most enriched TF in the community associated with a more healthy-like chromatin state and normal B-cell development [53]. Community 3 showed no difference in median accessibility signal, even though a small group of Pre-Treatment samples exhibited high signals. This suggests that this community might capture sites supporting malignant transformation specifically in this group of patients; this hypothesis is supported by the enriched CM and TF. EMT programs showed a prominent enrichment in this community. These programs are known to have a pivotal role in malignant transformation. Furthermore, the putative TF detected mostly overlapped with those enriched in community 1, which is dominated by sites involved in malignant transformation. Despite community 4 being the smallest community detected, its increased accessibility in Relapse samples made its analysis extremely valuable to define pathways involved in cancer recurrence. Interestingly, one of the enriched CM was related to malignant programs of neural progenitor cells, a pattern previously shown in several cancer types linked to developmental processes and stress [34] findings are supported by the TF enrichment analysis in this community, which returned TF involved in early development, such as NANOG and POU5F1 [54, 55]. Notably, SMAD3, a key component of the TGF-β signalling pathway, is also among the most enriched TF (Fig. 4D). This pathway has previously been implicated in B-ALL progression and immune evasion [56] in drug resistance of different cancer types [57–59]. Orthogonal analyses using Pscan-ChIP [60] confirmed the over-representation also at the level of the SMAD3 motif (MA0795.1; *P*-value = 0.0065).

Taken together, these observations underscore T-ChroNet’s ability to effectively mine patient-derived samples despite the high inter-sample heterogeneity, even in case of time points that are not close to each other. Furthermore, T-ChroNet successfully detected communities of accessible regions in which temporal trends, GO and TF are concordant with the alteration in the accessibility landscape carried by malignant transformation.

### T-ChroNet identifies communities that maximise interpretability and discovery

We then systematically compared the results of T-ChroNet against two widely used tools, one for network reconstruction from co-expression data, WGCNA [11] and one general-purpose clustering method, TCSeq (https://doi.org/10.18129/B9.bioc.TCseq). For TCSeq, we tested both hierarchical and *k*-means clustering. For both tools, we set parameters in a way that made them as comparable as possible to combining T-ChroNet with TChroNetR (see Methods).

Applied to the datasets from mouse liver development, TCSeq identified either two or three major clusters. With both clustering methods, one cluster captured the two T-ChroNet communities (2 and 3) enriched for signatures of hepatocytes (Supplementary Fig. S5B). The other one incorporated regions mainly from T-ChroNet communities 1 and 4. Overall, TCSeq failed at identifying clear subsets of regions strongly associated with the liver being a transient site of haematopoiesis during development (Supplementary Fig. S5B). The overall lower number of clusters identified also led to a lower number of upstream TF being enriched (Supplementary Fig. S6).

WGCNA was instead run with two different thresholds on the minimum number of regions per cluster, i.e. 100 or 1000. While for 100 we obtained 90 groups of regions, 1000 led to 7 clusters (Supplementary Fig. S7A). Due to the over-fragmentation of the first solution, we decided to explore only the results of the second one. Despite intermixing between the communities defined by T-ChroNet and the clusters obtained via WGCNA (Supplementary Fig. S7A), three of the clusters from WGCNA functionally matched those defined by T-ChroNet. More specifically, cluster 1 in part matched community 2 and 3 – showing enrichment for cell type markers (Supplementary Fig. S7B) and regulators (Supplementary Fig. S8) of the hepatocyte lineage. Cluster 3 instead showed similarity to community 1 – with enrichment for cell type markers (Supplementary Fig. S7B) and regulators (Supplementary Fig. S8), consistent with the liver being a transient site of haematopoiesis.

Taken together, these results show that while the conclusions extrapolated from running different methods are generally consistent, T-ChroNet with automatically tuned parameters provides an intermediate-grained view of the data, which enables a larger number of robust biological inferences from the obtained communities. TCSeq tend to capture only the major communities of co-accessible sites from the data, while WGCNA tend to over-fragment the data into a large number of smaller communities. This results in TCseq missing interesting, intermediate-sized communities. At the same time, the over-fragmentation from WGCNA reduces the power of the statistical analyses run to characterise the different communities. Besides, neither TCSeq nor WGCNA offer a fully automated option to fine-tune the parameters.

In our benchmark, WCGNA and TCseq failed to run on datasets larger than 60 000 regions (see Methods). Considering the range of dataset sizes that could be used for comparison (≤ 60 000), T-ChroNet compared favourably in terms of memory usage, with lower memory footprints (Supplementary Fig. S1B), while being at least as fast as WCGNA and TCseq (Supplementary Fig. S1A). On top of this, TChroNetPy showed more efficient multi-threaded implementations.

## Discussion

*Cis*-regulatory elements (CRE) are pivotal in regulating precise spatiotemporal gene expression. However, computational methods to dissect their temporal activity in development and malignant transformation remain limited. To fill this gap, we developed T-ChroNet, a network-based method to embed epigenomic data from temporal profiling and to detect communities of CRE with similar modulation patterns through time. T-ChroNet addresses the need for an accessible, general-purpose, fast, and flexible method to reconstruct and dissect networks of CRE.

To show the robustness and the applicability of T-ChroNet, we applied it to three publicly available datasets. Through the presented analysis, we demonstrated the capability of T-ChroNet to handle both ATAC-seq and histone ChIP-seq data, while successfully identifying communities showing distinct ontologies enrichment and specific subsets of TF that are supported by the literature, and allowing clear biological interpretation of each community. Notably, the application of T-ChroNet to the B-ALL datasets identified a relapse-specific community that we computationally associated with SMAD3. Notably, SMAD3 is implicated in the TGF-β signalling involved in B-ALL progression [56], and it was previously reported as a modulator of epithelial-mesenchymal transition and drug resistance in cancer [57–59]. Even though further investigations considering orthogonal and larger cohorts, along with functional validations, will be required, these findings point to a novel link between this signalling pathway and B-ALL relapse that could be potentially targeted.

Of note, using a non-parametric correlation measure allows full customization of the resulting network, also enabling the possibility to include other relevant covariates into the network. For instance, clinical data from patients can be embedded into the network to detect sets of regions that are highly associated with them, increasing the interpretability and the application of T-ChroNet to projects investigating malignant cell transformation.

T-ChroNet can also serve as a hypothesis generator tool. Through the identification of distinct, time-resolved communities of CRE showing coherent ontologies and upstream regulators, T-ChroNet enables the formulation of new testable hypotheses on the regulatory mechanisms of temporally-modulated cellular events. For instance, T-ChroNet communities can be used to prioritize subsets of CRE for CRISPR validation experiments.

On one hand, constructing networks based on Spearman’s rank correlation robustly captures monotonic relationships, is less sensitive to outliers and non-normal expression distributions, and allows straightforward *p*-value computation. On the other hand, one major limitation of T-ChroNet is that it intrinsically captures statistical similarities rather than regulatory causation. To this aim, several tools to estimate the activity of regulatory elements and to link them to their target genes are available. These can either use multi-omics bulk data (including but not limited to GRaNIE [15] and ANANSE [14]) or single-cell data (including but not limited to SCENIC+ [12], IReNA [16], and Pando [17]). More recently, methods such as Dictys [13] have attempted to build dynamic eGRN by partitioning cells along their inferred developmental trajectory, reconstructing context-specific networks. This represents the closest attempt to capture temporal regulatory modulation from single-cell data. While powerful in reconstructing eGRN, the goal of these tools is slightly different from that of T-ChroNet, and this comes at the expense of simplicity and interpretability. These methods also tend to rely on both chromatin accessibility and mRNA measurements, while also being particularly affected by false positives arising from the computational predictions of the binding of TF [18–20].

Another limitation of T-ChroNet is not being capable of distinguishing direct from indirect effects, along with a lack of directionality. Also, network-reconstruction tools based on pairwise statistical associations show limited performance on small or noisy datasets. Besides, at present they also lack ground truth, i.e. most of the validations of the inferred communities are based on enrichment approaches, or use other -omics data, rather than leading to functional validations. In the future, the availability of larger perturbation screens at the cell-type and cell-state resolution (e.g. Perturb-seq) could mitigate this. We expect that further development of T-ChroNET could partially overcome such limitations, at the expense of simplicity and interpretability. This would also include the use of mutual information–based measures. While more general, these would require larger sample sizes and would also introduce additional steps for the estimation of uncertainty (as compared to Spearman’s rank correlation, which allows straightforward *p*-value computation).

## Supplementary Material

lqag034_Supplemental_File

## Data Availability

The THP-1 H2K27ac counts were downloaded from the original publication by Reed *et al.* [28]. The mouse liver ATAC-seq bam and bed files are available in ENCODE with the following accession: ENCSR785NEL, ENCSR302LIV, ENCSR343TXK, ENCSR032HKE, ENCSR465PYP, ENCSR255XTC. The ATAC-seq data of the BCP-ALL cohort is available at are accessible at Gene Expression Omnibus (GEO) with accession number GSE214916. T-ChroNet and the code to perform the analyses shown in this paper are available at https://github.com/DGStefano/T-ChroNet and at http://zenodo.org/records/18878350, respectively.
