# Supplementary material for: T-ChroNet: Time-aware chromatin network reconstruction to detect dynamic regulatory programs in longitudinal epigenetic dataset
Source: NAR Genom Bioinform. 2026 Apr 8;8(2):lqag034. doi: 10.1093/nargab/lqag034 (PMC13069678; doi:10.1093/nargab/lqag034)
Supplement: lqag034_Supplemental_File [file lqag034_supplemental_file.pdf]

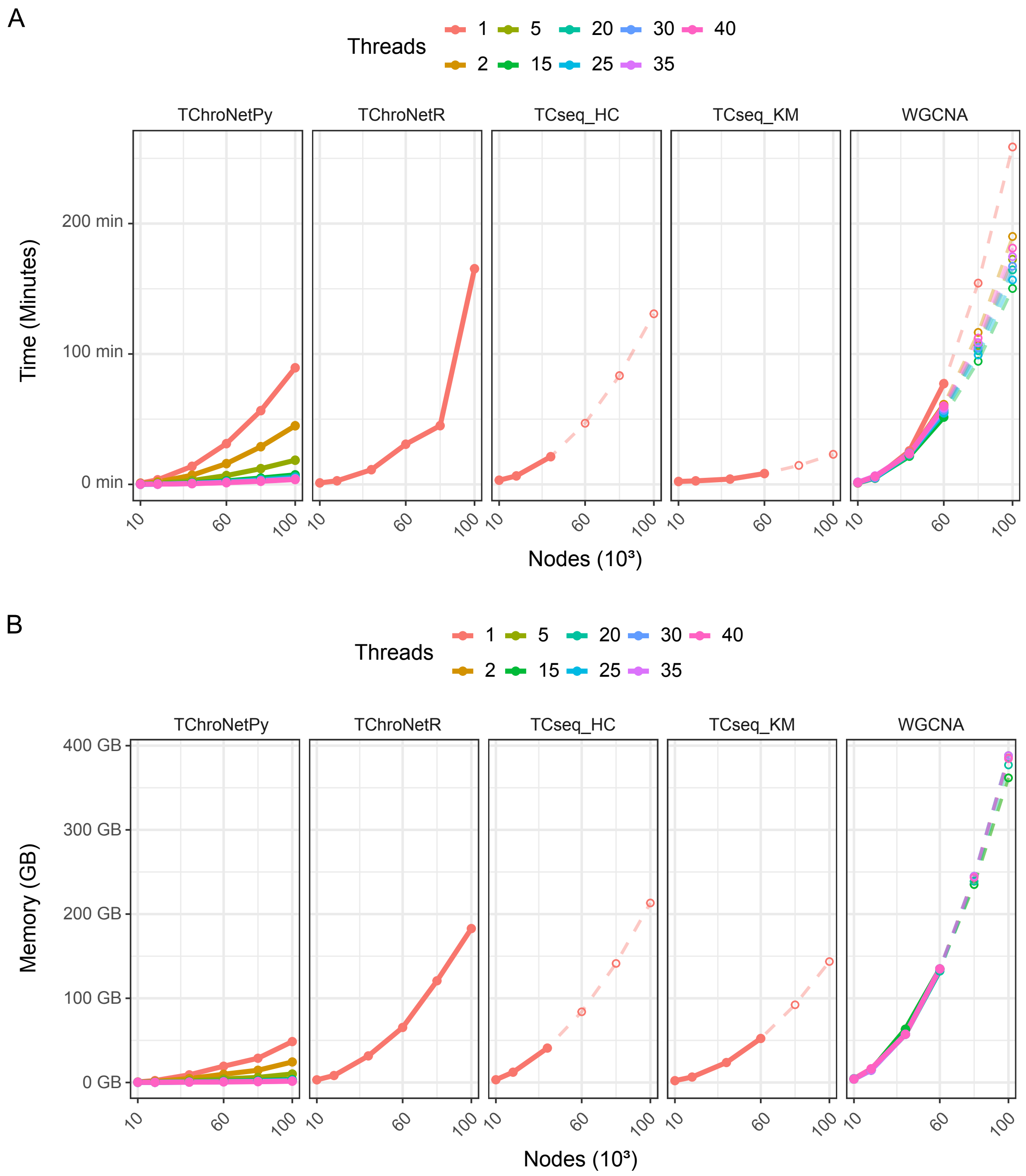

Supplementary Figure 1. (A) Line plots depicting the running performance of T-ChroNetPy, T-ChroNetR, TCSeq (hierarchical clustering and *k*-means clustering) and WGCNA, across different input sizes (10k to 100k nodes) and threads (as indicated by lines with different colours). The y-axis represents the running time in minutes, while the x-axis indicates the increasing number of nodes. The full dots connected by full lines indicate benchmark that were successfully run. Empty dots connected by dashed lines are instead estimated using a quadratic fit (based on the data points from the successful calculations), because of failure in running the benchmark with the indicated tool at the given number of nodes (see Methods). **(B)** Same as (A) but indicating memory consumption (Gb) at increasing number of nodes.

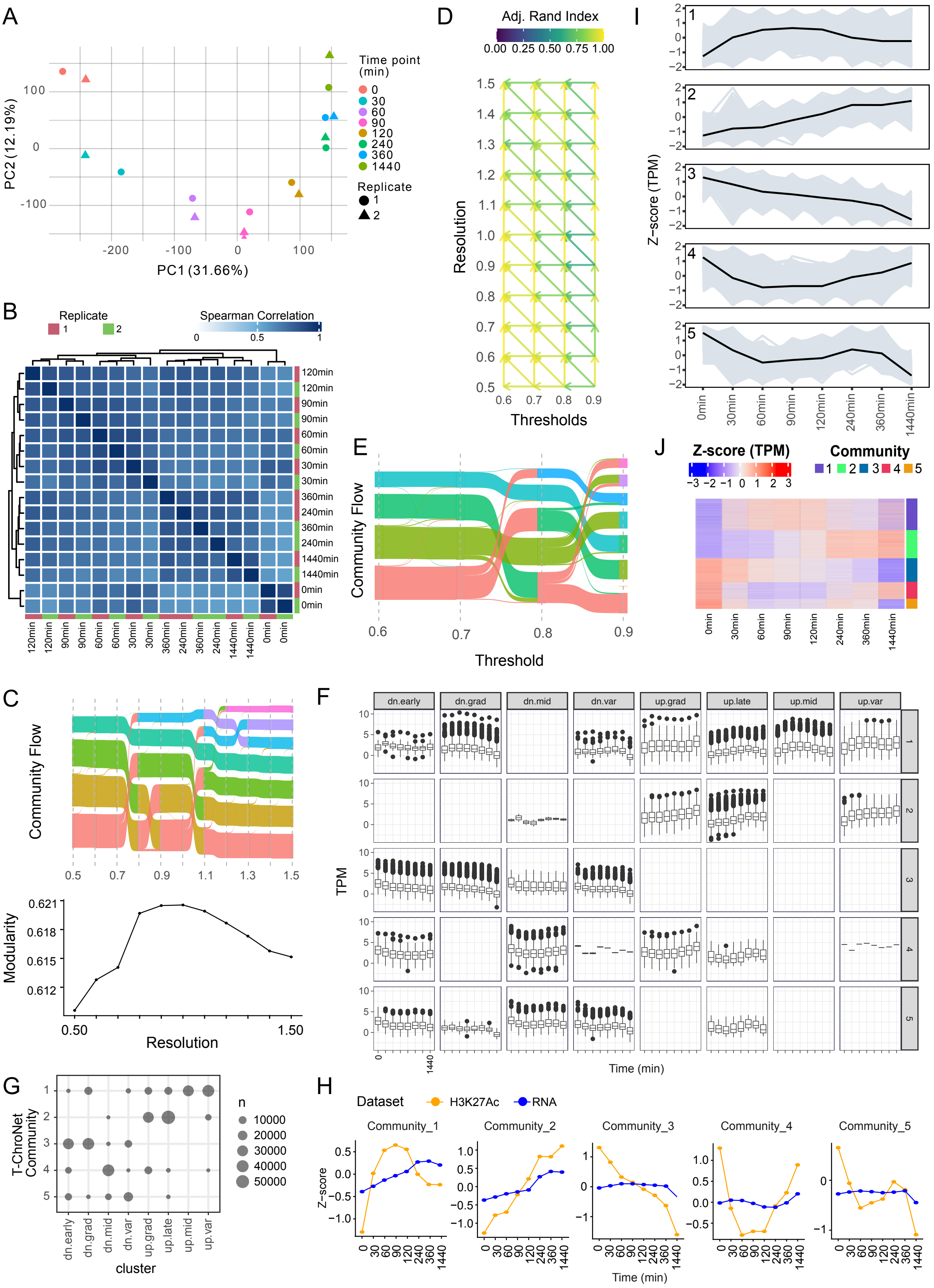

Supplementary Figure 2. (A) Principal Component Analysis (PCA) of samples according to the global H3K27ac profile after batch correction. **(B)** Heatmap showing the Spearman's rank correlation among all pairs of samples analysed. **(C)** (Top) Sankey plot considering the network at Spearman correlation's coefficient threshold of 0.8, illustrating the flow of

community structures across a range resolution parameters applied to the community detection algorithm. Each colour corresponds to a community detected at a given resolution, with band width proportional to the number of nodes in the community. Flows between adjacent resolutions indicate the proportion of nodes reassigned between communities as the resolution parameter changes. (Bottom) Line plot representing the modularity modulation (x-axis) at increased resolution parameters (y-axis). **(D)** Vector field showing the Adjusted Rand Index among communities detected at adjacent threshold and resolution values. Arrows connect resolution–threshold combinations being compared, and colour represents similarity, ranging from blue (low ARI) to yellow (high ARI). **(E)** Sankey plot for a fixed resolution (1.0) applied to the community detection algorithm, illustrating the flow of community structures when applying a rage of different thresholds to the Spearman's correlation coefficient during network reconstruction. Each colour corresponds to a community detected at a given resolution, with band width proportional to the number of nodes in the community. Flows between adjacent resolutions indicate the proportion of nodes reassigned between communities as the threshold on the Spearman's correlation coefficient is changed. **(F)** Box plot showing temporal trends in each cluster from the original paper (columns), faceted by T-ChroNet community (rows). **(G)** Dot plot showing the size of the subsets highlighted in (F). **(H)** Line plots depicting the temporal modulation of H3K27ac signal (orange) and gene expression of target genes (blue), separately for each community. Samples are ordered according to time. **(I)** For each community, line plots depict the temporal trend of peak expression. log2-normalized values are *z*-scored across samples; the black line represents the mean across peaks, and grey lines represent each peak of the community. Samples are temporally sorted. **(J)** Heatmap of *z*-scored, normalized reads' count, across communities. Individual replicates are showed, and temporally ordered.

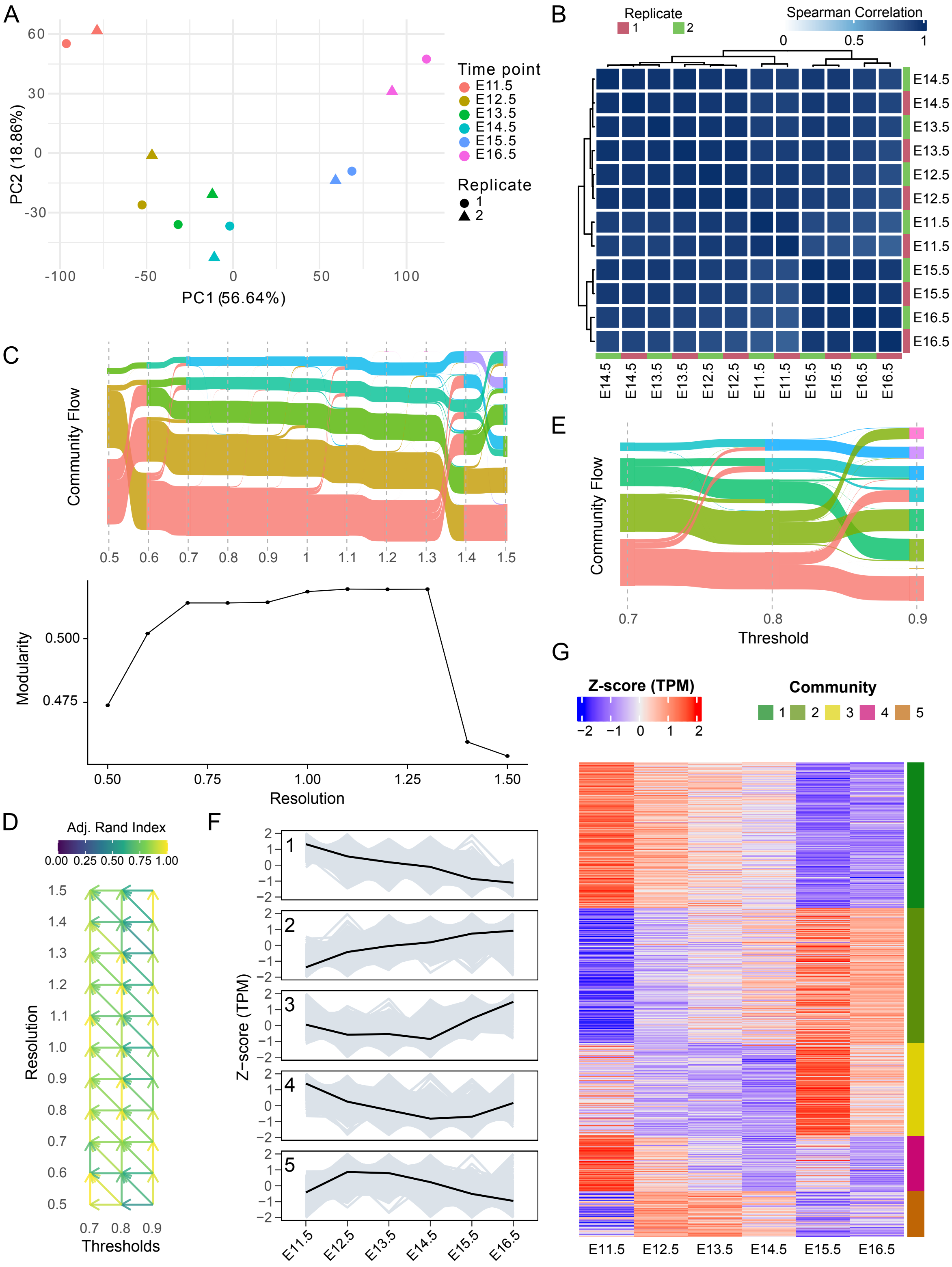

Supplementary Figure 3. (A) Principal Component Analysis (PCA) of samples according to the global chromatin accessibility profile after batch correction. **(B)** Heatmap showing the Spearman's rank correlation among all pairs of samples analysed. **(C)** (Top) Sankey plot considering the network at Spearman correlation's coefficient threshold of 0.8, illustrating the flow of community structures across a range resolution parameters applied to the community

detection algorithm. Each colour corresponds to a community detected at a given resolution, with band width proportional to the number of nodes in the community. Flows between adjacent resolutions indicate the proportion of nodes reassigned between communities as the resolution parameter changes. (Bottom) Line plot representing the modularity modulation (x-axis) at increased resolution parameters (y-axis). **(D)** Vector field showing the Adjusted Rand Index among communities detected at adjacent threshold and resolution values. Arrows connect resolution–threshold combinations being compared, and colour represents similarity, ranging from blue (low ARI) to yellow (high ARI). **(E)** Sankey plot for a fixed resolution (1.0) applied to the community detection algorithm, illustrating the flow of community structures when applying a rage of different thresholds to the Spearman's correlation coefficient during network reconstruction. Each colour corresponds to a community detected at a given resolution, with band width proportional to the number of nodes in the community. Flows between adjacent resolutions indicate the proportion of nodes reassigned between communities as the threshold on the Spearman's correlation coefficient is changed. **(F)** For each community, line plots depict the temporal trend of peak expression. log2-normalized values are *z*-scored across samples; the black line represents the mean across peaks, and grey lines represent each peak of the community. Samples are temporally sorted. **(G)** Heatmap of *z*-scored, normalized reads' count, across communities. Individual replicates are showed, and temporally ordered.

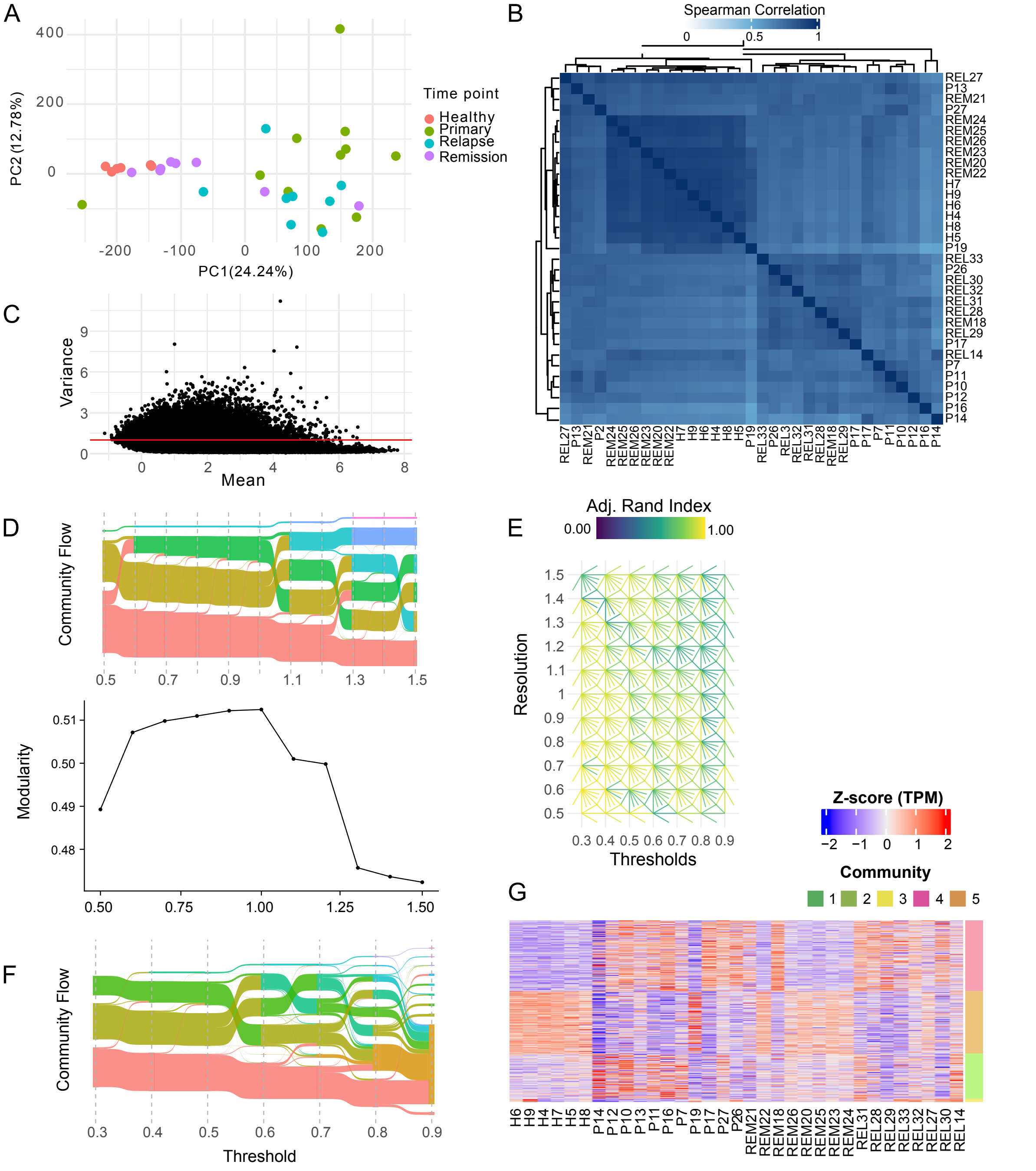

Supplementary Figure 4. (A) Principal Component Analysis (PCA) of samples according to the global chromatin accessibility profile after batch correction. **(B)** Heatmap showing the Spearman's rank correlation among all pairs of samples analysed. **(C)** Scatter plot illustrating the relation of mean and variance across all the samples for each detected region. The horizontal line indicates the threshold applied in order to select the variable region. **(D)** (Top) Sankey plot considering the network at Spearman correlation's coefficient threshold of 0.8, illustrating the flow of community structures across a range resolution parameters applied to the community detection algorithm. Each colour corresponds to a community detected at a given resolution, with band width proportional to the number of nodes in the community. Flows between adjacent resolutions indicate the proportion of nodes reassigned between communities as the resolution parameter changes. (Bottom) Line plot representing the

modularity modulation (x-axis) at increased resolution parameters (y-axis). **(E)** Vector field showing the Adjusted Rand Index among communities detected at adjacent threshold and resolution values. Arrows connect resolution–threshold combinations being compared, and colour represents similarity, ranging from blue (low ARI) to yellow (high ARI). **(F)** Sankey plot for a fixed resolution (1.1) applied to the community detection algorithm, illustrating the flow of community structures when applying a rage of different thresholds to the Spearman's correlation coefficient during network reconstruction. Each colour corresponds to a community detected at a given resolution, with band width proportional to the number of nodes in the community. Flows between adjacent resolutions indicate the proportion of nodes reassigned between communities as the threshold on the Spearman's correlation coefficient is changed. **(G)** Heatmap of *z*-scored, normalized reads' count, across communities. Individual replicates are showed, and ordered by stage of disease progression.

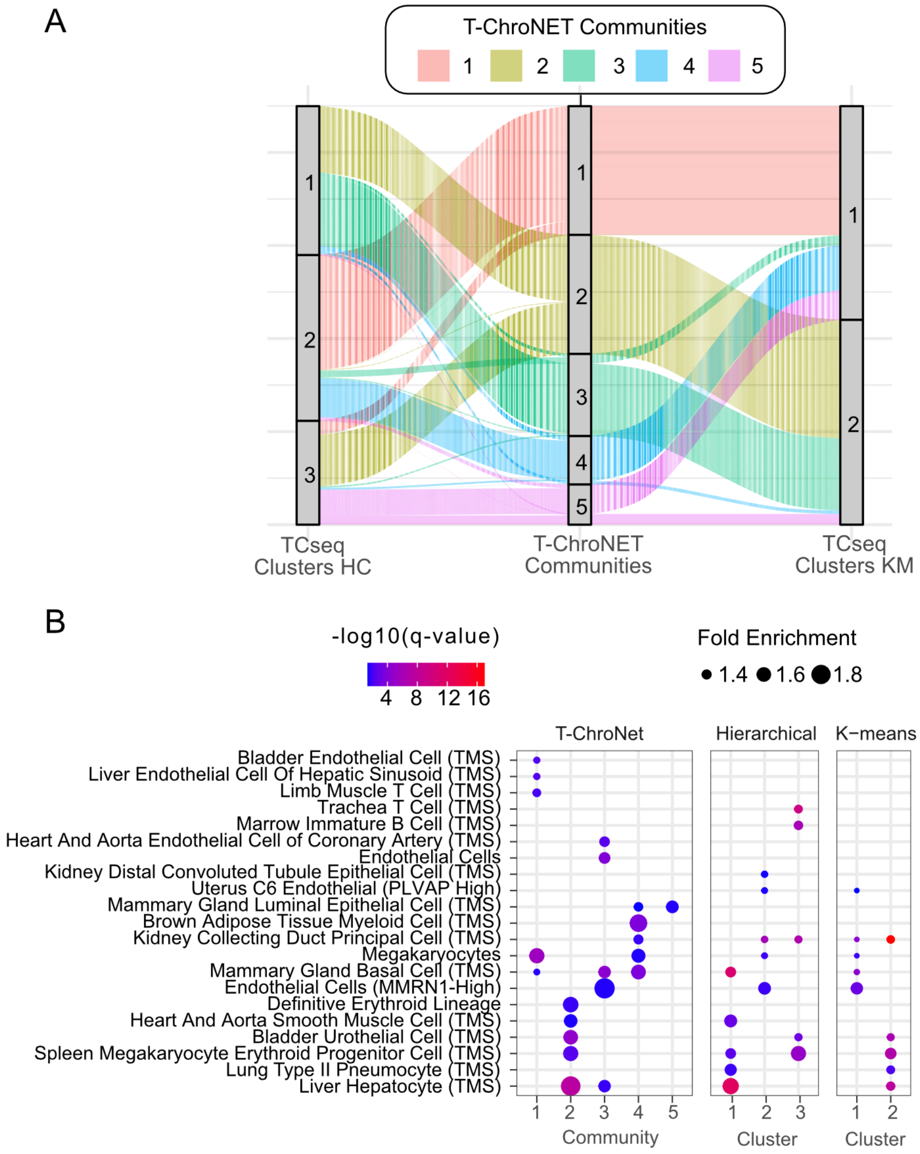

Supplementary Figure 5. (A) Sankey plot comparing the final communities obtained with T-ChroNet with those obtained with TCSeq using either hierarchical clustering (HC; left) or *k*-means clustering (KM; right). **(B)** Bubble chart representing the enrichment for markers of specific cell types (mouse MSigDB datasets) in the target genes of the CRE in each community, separately for the communities obtained with TCSeq (*k*-means and hierarchical, left) and T-ChroNet (right). Bubble size represents the fold enrichment, while the colour indicates the -log10(*q*-value) (red = high significance, blue = low significance; Hypergeometric test; Benjamini-Hochberg correction).

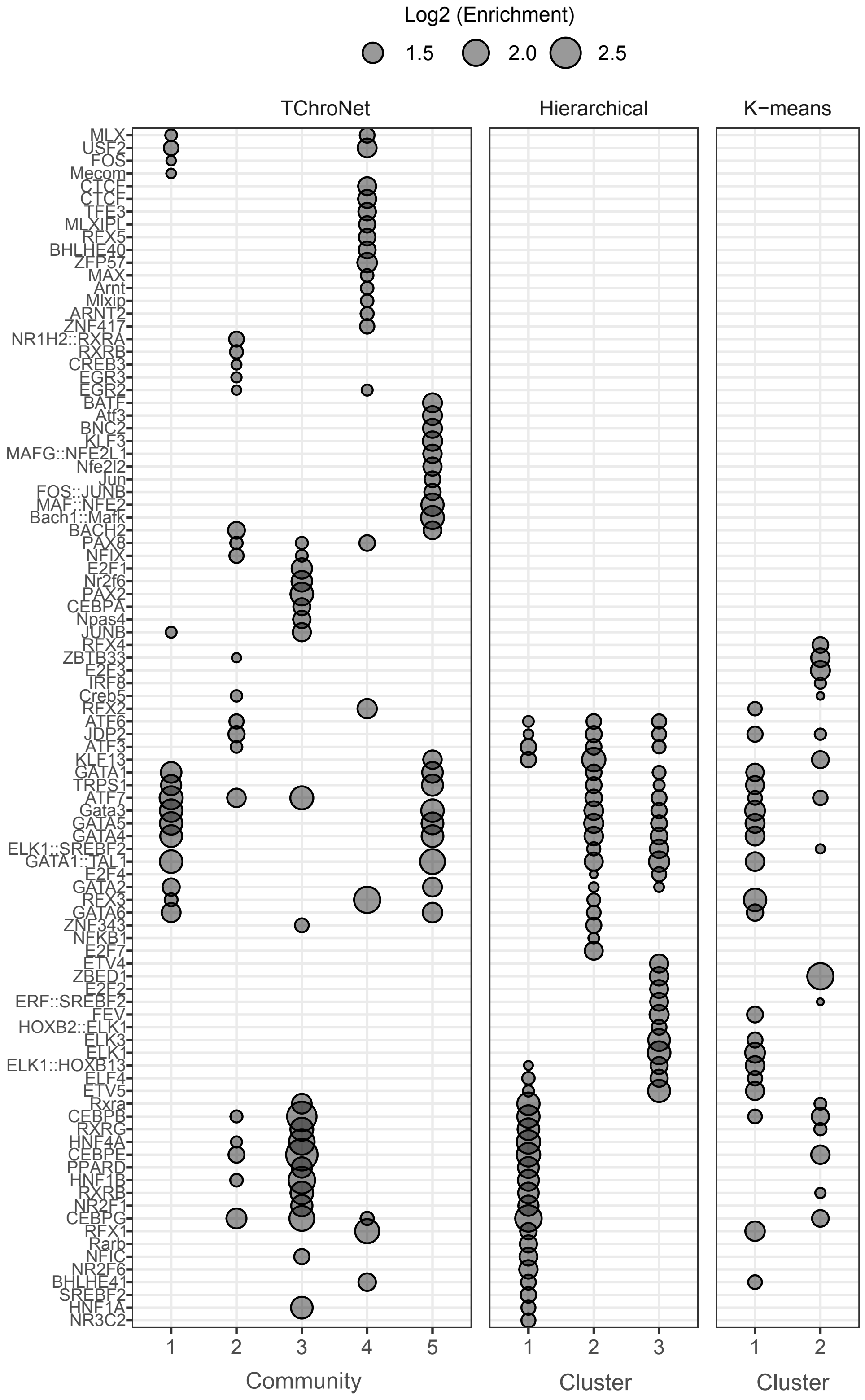

Supplementary Figure 6. (A) Bubble chart summarising the top five transcription factors (y-axis) enriched in each community (x-axis), separately for the communities obtained with TCSeq (*k*-means and hierarchical, left) and T-ChroNet (right). The size of the points indicates the Giggle Score.

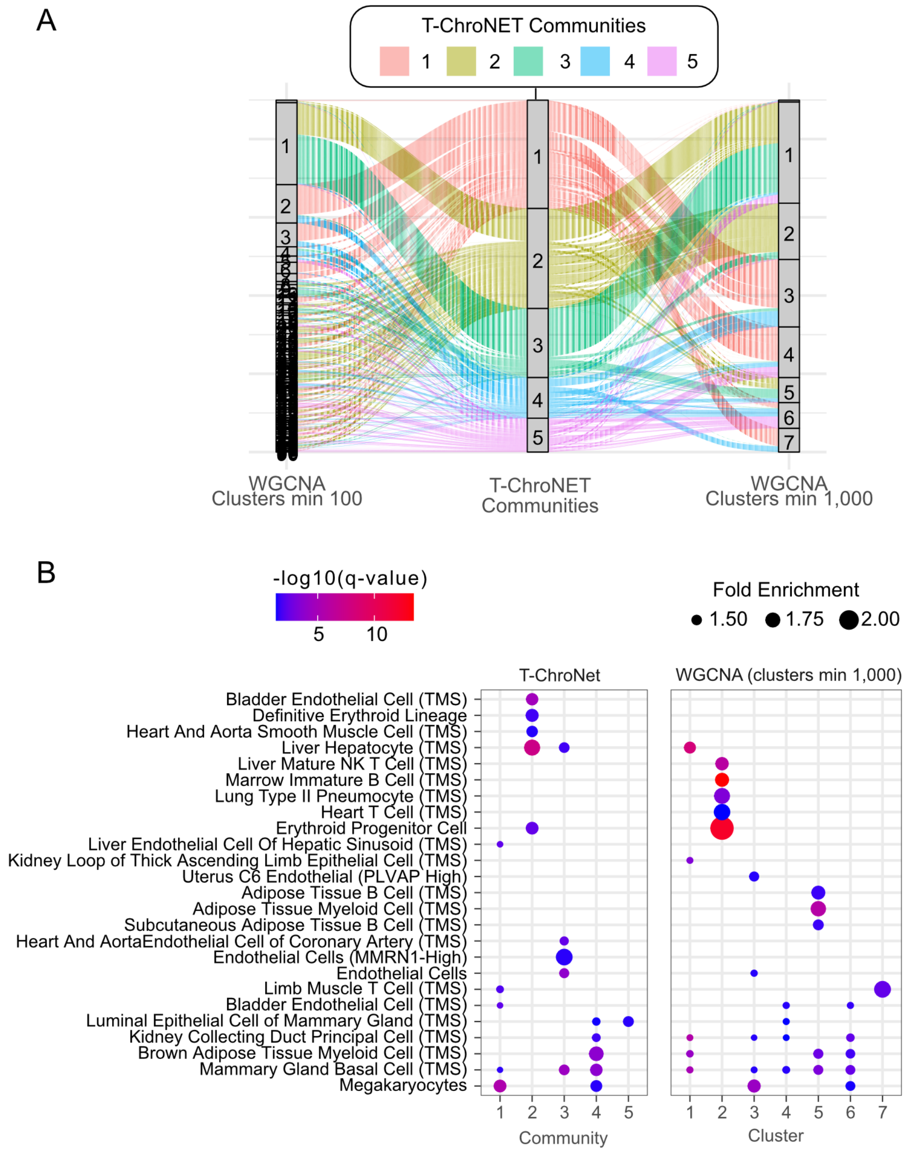

Supplementary Figure 7. (A) Sankey plot comparing the final communities obtained with T-ChroNet with those obtained with WGCNA considering a threshold on the minimum size of each cluster of 100 (left) or 1,000 (right). **(B)** Bubble chart representing the enrichment for markers of specific cell types (mouse MSigDB datasets) in the target genes of the CRE in each community, separately for the communities obtained with WGCNA (minimum size of each cluster of 1,000, left) and T-ChroNet (right). Bubble size represents the fold enrichment, while the colour indicates the -log10(*q*-value) (red = high significance, blue = low significance; Hypergeometric test; Benjamini-Hochberg correction).

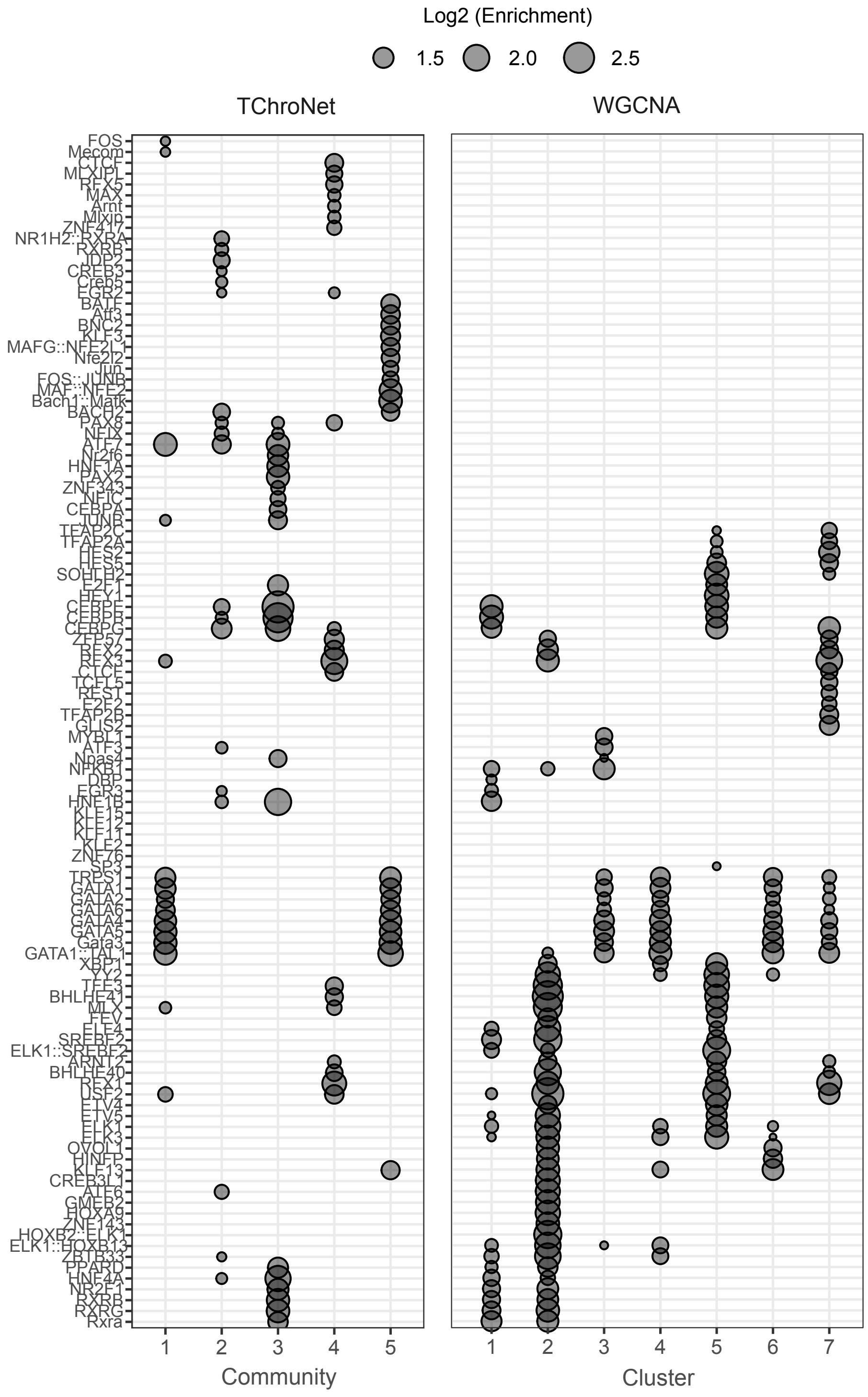

Supplementary Figure 8. (A) Bubble chart summarising the top five transcription factors (y-axis) enriched in each community (x-axis), separately for the communities obtained with WGCNA (minimum size of each cluster of 1,000, left) and T-ChroNet (right). The size of the points indicates the Giggle Score.
